# Live attenuated influenza viruses produced in a suspension process with avian AGE1.CR.pIX cells

**DOI:** 10.1186/1472-6750-12-79

**Published:** 2012-10-30

**Authors:** Verena Lohr, Yvonne Genzel, Ingo Jordan, Dietmar Katinger, Stefan Mahr, Volker Sandig, Udo Reichl

**Affiliations:** 1 Max Planck Institute for Dynamics of Complex Technical Systems, Sandtorstr. 1, 39106, Magdeburg, Germany; 2 ProBioGen AG, Goethestr. 54, 13086, Berlin, Germany; 3 Polymun Scientific GmbH, Donaustr. 99, 3400, Klosterneuburg, Austria; 4 University for Applied Sciences, Robert-Gerwig-Platz 1, 78120, Furtwangen, Germany; 5 Chair of Bioprocess Engineering, Otto-von-Guericke University Magdeburg, Universitätsplatz 2, 39106, Magdeburg, Germany

**Keywords:** Live attenuated influenza virus, Influenza vaccine production, Suspension cell culture, Cell density effect, AGE1.CR.pIX

## Abstract

**Background:**

Current influenza vaccines are trivalent or quadrivalent inactivated split or subunit vaccines administered intramuscularly, or live attenuated influenza vaccines (LAIV) adapted to replicate at temperatures below body temperature and administered intranasally. Both vaccines are considered safe and efficient, but due to differences in specific properties may complement each other to ensure reliable vaccine coverage. By now, licensed LAIV are produced in embryonated chicken eggs. In the near future influenza vaccines for human use will also be available from adherent MDCK or Vero cell cultures, but a scalable suspension process may facilitate production and supply with vaccines.

**Results:**

We evaluated the production of cold-adapted human influenza virus strains in the duck suspension cell line AGE1.CR.pIX using a chemically-defined medium. One cold-adapted A (H1N1) and one cold-adapted B virus strain was tested, as well as the reference strain A/PR/8/34 (H1N1). It is shown that a medium exchange is not required for infection and that maximum virus titers are obtained for 1 × 10^-6^ trypsin units per cell. 1 L bioreactor cultivations showed that 4 × 10^6^ cells/mL can be infected without a cell density effect achieving titers of 1 × 10^8^ virions/mL after 24 h.

**Conclusions:**

Overall, this study demonstrates that AGE1.CR.pIX cells support replication of LAIV strains in a chemically-defined medium using a simple process without medium exchanges. Moreover, the process is fast with peak titers obtained 24 h post infection and easily scalable to industrial volumes as neither microcarriers nor medium replacements are required.

## Background

Annual vaccination is the most efficient way to reduce the immense number of 3–5 million cases of severe illness or death due to influenza virus infections. Different vaccine types have been developed and are being deployed for this purpose over the past 60–70 years. The most common type is the trivalent influenza vaccine (TIV) derived from inactivated whole virus. TIVs are injected intramuscularly or intradermal. They have a favorable safety profile and induce reliable protective immunity against viral strains which are similar to those present in the vaccine. However, because efficacy against new virus variants can be low, the composition of the vaccine has to be carefully predicted for each influenza season [1]. Another type of influenza vaccines is the live attenuated influenza vaccine (LAIV) comprising virus strains that are adapted to replication at temperatures below the human body temperature. LAIVs are administered through a nasal spray mimicking a natural infection of the mucosal cells of the nasopharynx. However, these viruses cannot replicate at non-permissive temperatures present in the lower respiratory tract and lungs. Due to the attenuated infection in the upper respiratory tract, these vaccines induce not only strain specific immunity but also T cell responses that may be cross-reactive and thus contribute to protection against a wider spectrum of influenza A and B strains [2-4]. The only approved LAIVs are FluMist® (MedImmune) for the US and Russian market and, since 2011, the European analogue Fluenz®, both produced in an egg-based process [5]. As opposed to parenteral inactivated vaccines, the downstream processing requirements are less stringent and thus less expensive for mucosal live virus vaccines. Also for this reason, the WHO encouraged to extend the manufacturing of LAIVs as part of the global pandemic influenza action plan [6].

Production of LAIVs has so far been investigated in three host systems: chicken eggs [7], adherent MDCK [8-10] or adherent Vero cells [11-13]. Because properties such as replication kinetics, viral RNA synthesis and protein expression appear to be comparable in cell culture- or egg-derived virus strains [14], both have been approved for the production of vaccines for human use. However, there are inherent limitations associated with the process design related to the decision for a specific host system, being more or less pronounced for one or the other. Scalability, for example, is impaired significantly for adherent cell lines cultivated in static or microcarrier systems. The use of suspension-adapted cell lines such as MDCK and Vero could overcome this limitation. These cells have been adapted to growth in suspension by several groups [15-18] and by the industry where they are already used in vaccine manufacturing [19]. Other suspension cell lines useful for influenza vaccine production are PER.C6 [20], HEK293 [21], EB66 [22] or AGE1.CR.pIX (in the following: CR.pIX) [23,24].

The CR.pIX cell line has been generated specifically for production of modern vaccines. Its derivation and identity is fully documented and genetic stability was shown for at least 90 passages [25]. As required by regulatory authorities, the absence of adventitious agents was confirmed via extensive testing of CR.pIX cell banks by a spectrum of methods such as transmission electron microscopy against microbial structures; diagnostic PCR reactions against specific avian, bovine and porcine agents; in vitro assays with various sentinel cell lines for any cytopathic effects; in vivo assays in different animal species for any signs of infection or induction of antibodies; quantitative fluorescent product enhanced reverse transcriptase (QFPERT) assay and co-cultivation to detect endogenous or exogenous retroviral contamination; and inoculation of cell line material into various agars and broths to test against mycoplasma and other bacteria. With regards to process technology, this cell line proliferates in suspension in a chemically-defined medium and has been shown to support influenza A virus replication. Moreover, CR.pIX cells are expected to be susceptible for a broad spectrum of virus subtypes and strains as they display both α2,3- and α2,6-linked sialic acids as surface membrane receptors [26].

For the design of vaccine production processes using a new host cell, studies concerning crucial infection parameters like trypsin activity and multiplicity of infection (moi) are necessary. In addition, the cell concentration at time of infection (CCI) has been reported to be important for adenovirus, baculovirus and influenza virus production [21,27-30]. These studies demonstrated that above a certain CCI, the cell-specific virus productivity drops substantially, what is called the “cell density effect”. All studies related to this topic indicate that the reasons for the cell density effect are highly dependent on the production system (mainly virus species, host cell line and medium) and thus cannot be generalized. Explanations for this effect include depletion of nutrients or accumulation of inhibiting substances [30]. Also, activity or availability of host cell factors required for virus replication may change with the cell cycle [31] or with metabolic shifts [28,31] at higher cell densities. As an example, for production of influenza virus typically 1-2 × 10^6^ cells/mL are used, but with fed-batch or perfusion strategies exhaustion of medium components and influence of inhibiting substances can be avoided and high-yield infections of 1 × 10^7^ cells/mL have been demonstrated [32,33]. The aim of overcoming the cell density effect is to maintain or even increase the cell-specific productivity in order to improve process productivity, i.e. to increase the vaccine doses which can be harvested per reactor volume.

In this work, we produced human LAIV strains in a chemically-defined medium using CR.pIX cells. One cold-adapted (*ca*) influenza A strain (H1N1), one *ca* influenza B strain as well as the reference strain A/PR/8/34 (H1N1; not cold-adapted) were propagated. After adapting the virus strains to CR.pIX cells we confirmed temperature sensitivity and identified best cultivation conditions such as medium supply and trypsin activity for successful infection in small-scale scouting experiments. Subsequently, batch bioreactor cultivations were performed to demonstrate that cultures can be productively infected at a CCI of 4 × 10^6^ cells/mL without any medium exchange, doubling the CCI that has been used previously with CR.pIX cells (2 × 10^6^ cells/mL) [23]. Only at higher CCI a cell density effect was observed when infecting without a medium exchange. In all experiments, high titers and fast replication dynamics have been achieved demonstrating a chemically-defined suspension production process for influenza LAIV strains.

## Results

### The chemically-defined medium CD-U2 supports direct infection

Experimental data on influenza virus production in CR.pIX cells is available only for cultures in serum-free proliferation medium containing hydrolysates [23]. However, to improve proliferation of CR.pIX cells and to reduce risk of lot-to-lot inconsistencies due to variable hydrolysate composition, a chemically-defined medium (CD-U2) has been developed specifically for this cell line [25]. As a reference for the evaluation of cold-adapted virus propagation, and to test virus stability and the requirement for a medium exchange, replication of the human influenza virus strain H1N1 A/PR/8/34 was investigated first. This strain is included regularly in similar studies and titers obtained with CR.pIX cells therefore can be compared to other producer cells.

CR.pIX cells were infected with A/PR/8/34 without medium exchange, after a partial medium exchange (half of the culture), or after a complete medium exchange. Infection took place at a CCI of 2 × 10^6^ cells/mL that was reached approximately two days after culture inoculation.

**Figure 1:**
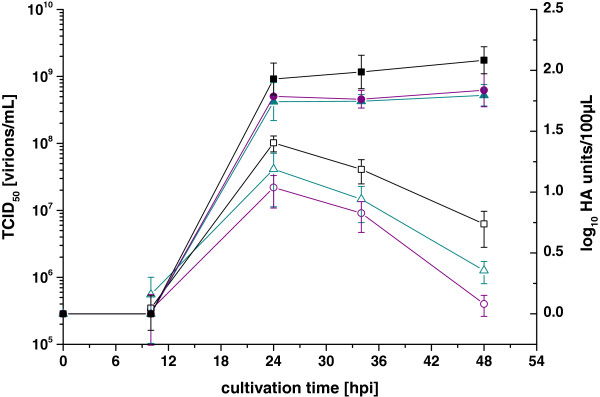
**TCID**_**50**_**titers (open symbols) and HA yields (closed symbols) from infections performed with different medium exchange strategies.** CR.pIX cells were infected with influenza strain A/PR/8/34 in shaker flasks at a CCI of 2 × 10^6^ cells/mL and 1 × 10^-6^ units trypsin per cell. Three strategies were performed: infection without medium exchange (■, black), after a medium exchange (▲, cyan) and after the exchange of 50% of the medium (●, purple). Error bars indicate standard deviation of three independent cultures.

HA yields and TCID_50_ titers differed slightly using the three medium exchange strategies (Figure 1) with a trend towards lower titers when a medium exchange was applied. The HA yield reached a plateau of 2.0-2.3 log_10_ HA units/100 μL at 24 h post infection (hpi) and remained stable until 72 hpi (later data points not shown). TCID_50_ titers reached a maximum at 24 hpi, but were less stable than the HA yield, decreasing about 1 log unit in 48 h. The maximum TCID_50_ of 1.3 × 10^8^ virions/mL was obtained in cultures which have been infected directly and lowest TCID_50_ was observed after exchanging half of the growth medium. None of the cultures exhibited limiting substrate levels at time of infection, which was confirmed with metabolite concentration measurements (data not shown). From these results we concluded that a medium exchange is not required and that CD-U2 supports stable HA virus yields and good TCID_50_ titers at a CCI of 2 × 10^6^ cells/mL.

### Temperature sensitivity of LAIV strains is maintained after adaptation

Influenza virus has a broad host range and can shuttle between mammalian and avian cells. However, virus replication dynamics and maximum titers may be impaired by a sudden shift to a new cell substrate and may be improved by serial passages of the virus in the new host cell line. For this reason, the LAIV strains A/Singapore and B/Vienna (for details on these strains see materials & methods section) were adapted to CR.pIX cells to generate working seed viruses with high titers. At the same time, care was taken to achieve this with a minimum number of passages (maximum 3) to minimize the potential for reversion to replication at non-permissive temperatures.

After adaptation to CR.pIX cells, the A/Singapore and B/Vienna strain had, respectively, 1.1 and 1.4 log_10_ increased maximum TCID_50_ titers. The chosen seed viruses were from passage 3 for the A/Singapore strain, harvested at 28 hpi, and from passage 2 for the B/Vienna strain, harvested at 34 hpi. Overall, 5 (A/Singapore) and 6 days (B/Vienna) were needed to complete the adaptation to CR.pIX cells. The mentioned harvests were used as working seed viruses for all subsequent experiments.

Considering temperature sensitivity, we tested replication of both *ca* strains at 37°C before adaptation to CR.pIX cells and could not detect any virus titer, whereas virus propagation was successful at 33°C (Table 1). To test whether the required temperature sensitivity of LAIV strains was still present after the adaptation process, infection experiments were conducted at 33, 37 and 40°C. The B strain was highly sensitive to non-permissive temperature as no viral activity was determined at 37°C, obviating further examination of replication at 40°C. The A/Singapore strain produced about 40 times less active virus particles at 37°C compared to 33°C. At 40°C, no active particles were detected. These results indicate that both working virus seeds exhibit the expected temperature sensitivity.

**Table 1:** Temperature sensitivity of A/Singapore and B/Vienna viruses in CR.pIX cells

**Maximum TCID**_**50 **_titers [virions/mL]^*^	**33°C**	**37°C**	**40°C**
Before adaptation to CR.pIX cells			
A/Singapore	1.3 × 10^8^	0	-^#^
B/Vienna	1.8 × 10^5^	0	-
After adaptation to CR.pIX cells			
A/Singapore	2.4 × 10^8^	1.8 × 10^6^	0
B/Vienna	3.2 × 10^6^	0	-

^*^Maximum titers of CR.pIX cells infected in T-flasks at a CCI of 2 × 10^6^ cells/mL and with 1 × 10^-6^ units trypsin per cell.

^#^not determined.

### Optimization of trypsin activity

**Figure 2:**
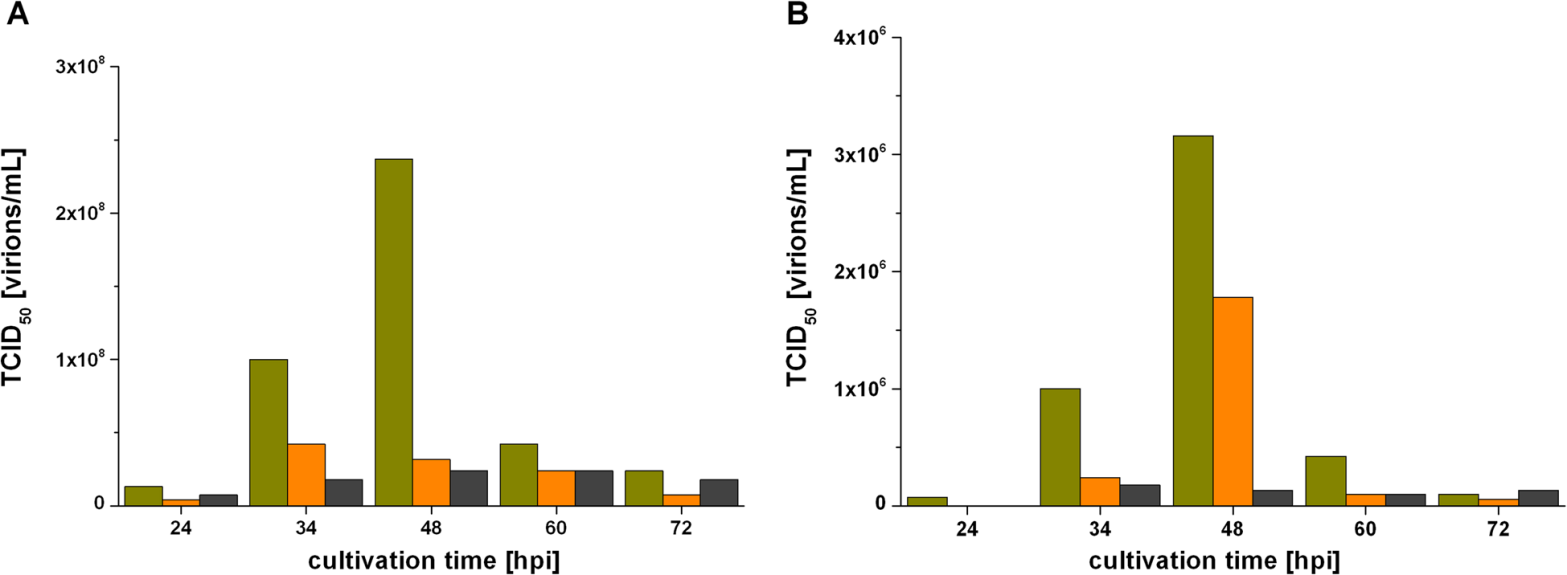
**Optimization of trypsin activity used for the infection of CR.pIX cells with LAIV strains A/Singapore (A) and B/Vienna (B).** Tested trypsin activities were 1 × 10^-6^ units/cell (green), 5 × 10^-6^ units/cell (orange) and 10 × 10^-6^ units/cell (black). Cells were infected in T-flasks at a CCI of 2 × 10^6^ cells/mL without applying a medium exchange.

LAIV strains replicate at 33°C and the activity of trypsin (initially optimized for replication at 37°C) is expected to be slightly reduced at 33°C [34]. To test whether this reduction requires higher amounts of trypsin for propagation of cold-adapted virus strains, we tested different trypsin activities with A/Singapore and B/Vienna (Figure 2). When using 1 × 10^-6^ units/cell (standard concentration for A/PR/8/34 propagation), maximum TCID_50_ values were measured at 48 hpi. An increase of the trypsin activity to 5 × 10^-6^ units/cell or even 10 × 10^-6^ units/cell reduced the titers of A/Singapore and B/Vienna. Infections without trypsin addition did not produce any infectious virus particles. Thus, without further adaptation of this parameter, 1 × 10^-6^ units trypsin per cell was found to support best the replication of A/Singapore (2.4 × 10^8^ virions/mL) and B/Vienna (3.2 × 10^6^ virions/mL).

### Cell density effect in bioreactor cultivations

**Figure 3:**
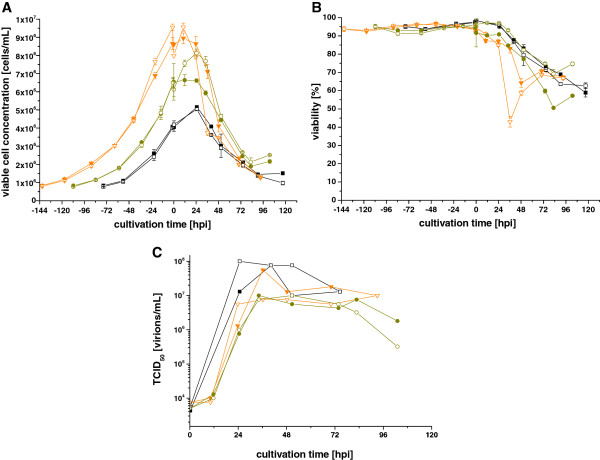
**Infection of CR.pIX cells with the LAIV strain A/Singapore at different cell concentrations at time of infection.** Viable cell concentrations **(A)**, viabilities **(B)** and TCID_50_ titers **(C)** of bioreactor cultivations are shown. Cells were infected with parameters summarized in Table 2: CCI_4_1 (■, black) and CCI_4_2 (□, black), CCI_6_1 (●, green) and CCI_6_2 (○, green), CCI_8_1 (▼, orange) and CCI_8_2 (∇, orange). After the cell growth phase at 37°C, bioreactor temperature was controlled at 33°C during the infection phase. Error bars for viable cell concentrations and viabilities indicate standard deviations of triplicate measurements.

Whether the *ca* strains can be produced in bioreactors is an important test towards suitability of LAIV strain production for seasonal or pandemic vaccines in the CR.pIX suspension cells. Focusing on the A/Singapore strain, we therefore performed experiments in a benchtop stirred tank bioreactor system to test the scale-up potential. The cell density effect was evaluated by varying the cell concentration at time of infection with the aim to identify the highest CCI that can be used without reduction of cell-specific productivity. One problem for infection at high CCI could be the release and accumulation of trypsin inhibitors during the growth phase. We therefore also tested if a higher trypsin activity or a medium feed at high CCI can rescue productivity. Six bioreactor runs were performed with infections at 4 × 10^6^ cells/mL (three days batch growth), 6 × 10^6^ cells/mL (five days batch growth) and 8 × 10^6^ cells/mL (six days batch growth) (Figure 3A). Exact cell concentrations together with added trypsin and medium are summarized in Table 2.

**Table 2:** Bioreactor cultivations performed with the LAIV strain A/Singapore

	**CCI_4_1**	**CCI_4_2**	**CCI_6_1**	**CCI_6_2**	**CCI_8_1**	**CCI_8_2**
CCI [viable cells/mL]	4.2 × 10^6^	4.1 × 10^6^	6.5 × 10^6^	6.1 × 10^6^	7.4 × 10^6^	8.0 × 10^6^
Trypsin conc. [units/cell]	10 × 10^-6^	1 × 10^-6^	1 × 10^-6^	1 × 10^-6^	10 × 10^-6^	10 × 10^-6^
Feed	-	-	-	-	-	200 mL

CR.pIX cell cultures were infected at different CCI and trypsin activities. Upon infection, the temperature was reduced to 33°C for virus propagation.

At the lowest CCI (4 × 10^6^ cells/mL), cell-specific virus yields of 15 (CCI_4_1) and 19 active virions/cell (CCI_4_2) were obtained. Infection of the intermediate group with a CCI of about 6 × 10^6^ cells/mL led to reduced titers (1 × 10^7^ virions/mL for both cultures) compared to the lowest CCI (1 × 10^8^ virions/mL) and, correspondingly, reduced cell-specific productivities (1–2 virions/cell). Using a high CCI of about 8 × 10^6^ cells/mL resulted in titers of 8 × 10^6^ virions/mL and 6 × 10^7^ virions/mL, respectively, and hence also low cell-specific virus yields of 6 and 2 virions/cell, respectively. This shows that neither the titer nor the productivity was rescued when applying a higher trypsin activity (10 × 10^-6^ units/cell) or a medium addition (200 mL) at the highest CCI. For all cultures, viability at time of infection was above 90% (Figure 3B), showing that cells were not impaired due to extension of the growth phase. Thus, a cell density effect, i.e. a loss in cell-specific virus productivity, was observed at CCI above 4 × 10^6^ cells/mL.

A comparison to shaker flask experiments (which resulted in 15–20 virions/cell, data not shown) confirmed that scale-up to a benchtop bioreactor did not reduce virus titers or productivities at a CCI of 4 × 10^6^ cells/mL. From these results we conclude that a CCI of up to 4 × 10^6^ cells/mL can be used for virus production without the requirement for a medium exchange and, most importantly, without loss of cell-specific productivity.

## Discussion

Increasing and unmet demand for vaccination against seasonal and pandemic influenza virus is a strong motivation for exploring additional means in vaccine manufacturing. Currently, most inactivated and the live attenuated influenza vaccines are manufactured in embryonated chicken eggs, an established technology but associated with demanding logistics, risks of allergic reactions and difficulties in scalability. Therefore, some influenza vaccine manufacturers have developed new production processes for influenza vaccines in cell culture (Vero and MDCK cells), although LAIVs are still exclusively produced in eggs [35]. In particular, the use of continuous cell lines adapted to growth in suspension has the potential for large scale production in closed systems using a comparatively simple generic process. We therefore investigated replication of influenza virus in the CR.pIX cell line, which is specifically created and designed for vaccine manufacture and is adapted to proliferate in chemically-defined media. Generally, CR.pIX cells show a good proliferation performance with growth rates in the range of 0.016 and 0.023 h^-1^ (derived from the exponential growth phase of the bioreactor cultivations shown in Figure 3). After seven days, approximately 1 × 10^7^ cells/mL can be achieved in batch cultivation. In conjunction with a well-balanced medium (CD-U2), this leads to a short growth phase so that infections can be initiated soon after inoculation.

In the first part of our study we demonstrate that CR.pIX cells can produce influenza virus to high titers in shaker flasks. For the reference strain A/PR/8/34, TCID_50_ titers of 1.3 × 10^8^ virions/mL were achieved, which is in a similar range (10^7^ to 10^9^ virions/mL) reported for other cell lines [19,35,36]. The achieved HA titers (used to calculate the number of vaccine doses for inactivated virus vaccines) are about 2.0 log HA units/100 μL which is slightly lower than those normally obtained with conventional cells (MDCK or Vero cells) or new candidate cell lines (HEK293, PER.C6). Mostly, these are reported to propagate the A/PR/8/34 influenza virus strain to titers of 3.0 to 3.6 log HA units/100 μL [21,35-37]. Generally, CR.pIX cells seem to generate the same TCID_50_ titers, but slightly lower HA titers and thus should in particular be considered for the production of LAIV strains. With a medium replacement at time of infection, we could not observe a titer increase and thus we conclude that a process without any medium exchange is possible in this system, a property which substantially simplifies a suspension process at industrial scales.

LAIV strains must have a temperature-sensitive and cold-adapted phenotype in order to be considered safe for use in human vaccination. Thus, we tested temperature sensitivity after adapting the *ca* strains to CR.pIX cells. The B/Vienna strain showed to be temperature-sensitive as no active virions were produced at 37°C. The titer of A/Singapore was reduced about 1.5 log at 37°C compared to 33°C (40%). This reduction is not as clear as for the B strain but expected from previous studies as the replication deficiency must be obvious only at 39 or 40°C for A strains [38]. Other studies have demonstrated a titer reduction of 20% at 37°C compared to the titer at 33°C [39] and of 2 log units for the master donor strain B/AnnArbor/1/66 strain [40]. This range was also observed here. Testing the temperature sensitivity before and after adaptation to CR.pIX cells (restricted to 2–3 passages) confirmed the temperature-sensitive phenotype. Both *ca* strains were pheno- and genotypically characterized for patent registration before reassortment and adaptation to CR.pIX cells [41], and it was shown that both cold-adapted strains carry several mutations compared to the wild type strains. For the A/Singapore strain there were 13 mutations with 8 of them coding for an amino acid exchange. For the B/Vienna strain 5 mutations were detected by sequencing with 3 of them coding for an amino acid exchange. Generally, sequencing of both strains could be of interest for follow-up studies considering mutations that occur during adaptation to the host cell line CR.pIX and considering the presence of mutations that might be connected with the temperature-sensitive phenotype [40,42,43].

It is known that for optimal influenza virus replication trypsin addition is crucial. The selection of the trypsin activity depends on the cell line (depending on proteases that are secreted), the medium (protein content), and also the virus strain [44]. Here, for both LAIV strains we tested 3 different concentrations to identify an optimal trypsin activity and found 1 × 10^-6^ units/cell to result in highest titers. The reduced temperature of 33°C did not have an effect on the optimal trypsin concentration due to an only slightly impaired enzyme activity at this temperature [34]. However, a design of experiment approach considering trypsin activity and other parameters would be useful for further process optimization [17]. Shaker flask experiments were found to be highly predictive and scale-up to stirred tank bioreactors was possible without further optimization and without loss of productivity. Particularly this easy scale-up provides higher flexibility compared to eggs or adherent cells.

A cell density effect, i.e. reduced titers and productivities at high CCI, as described for HEK293 cells and insect Sf9 cells infected with adenovirus, influenza virus and baculovirus [21,27,29,30,45], was also observed with influenza virus-infected CR.pIX cells. At CCI above a critical concentration of 4 × 10^6^ cells/mL, cell-specific virus productivity decreased. The same critical cell concentration (4 × 10^6^ cells/mL) was also found in studies using A/PR/8/34-infected HEK293 cells [21]. Influenza virus replication is known to modulate the cell cycle to include the cellular environment for replication [46]. We have observed previously that the cell cycle fractions of a CR.pIX population at 8 × 10^6^ cells/mL (six days growth) do not differ significantly from the fractions at 2 × 10^6^ cells/mL, which corresponds to about three days growth (data not shown). For this reason, we focused on medium limitations as a cause for the cell density effect. Addition of a 10-fold higher trypsin activity suggests that impaired virus propagation was not due to trypsin inhibitors released by the cells during the growth phase. Furthermore, the depletion of a common medium component also appears unlikely as a medium feed could not rescue virus titers or cell-specific productivities. Supporting this observation, neither the concentrations of the tested amino acids nor metabolites such as glucose or pyruvate appeared to be limiting (data not shown). Still, we cannot rule out that the accumulation of more elusive medium compounds (for example lipids, trace elements or vitamins) may be responsible. Thus, at this moment we speculate that accumulation of inhibiting substances (that we did not measure) may cause the cell density effect. This hypothesis is currently tested in perfusion cultivations where cells are infected at very high cell concentrations. Future studies will also investigate modification of the medium (which was designed for proliferation and compatibility with a biphasic poxvirus production process [25]) to increase not only virus titers and productivity but also the critical CCI.

Our results show that the *ca* strain A/Singapore can replicate to 1 × 10^8^ virions/mL in a CR.pIX cell-based process. This corresponds to approximately 10000 vaccine doses per liter reactor volume assuming that one dose contains 10^7^ active virus particles (not taking into account losses during downstream processing). Romanova et al. obtained 2 × 10^8^ pfu/mL with the same master donor strain (A/Singapore/1/57 *ca*) using adherent Vero cells [11]. With adherent MDCK cells, titers for the related strain A/NewCaledonia/20/99 of up to 10^9^ FFU/mL have been reported [8,10,14,47]. Several influenza B strains were also tested in those studies and were produced to titers of 10^8^ to 10^9^ virions/mL. As it is generally known that achievable titers are strain-specific, extensive optimizations normally help to maximize titers for each strain. Aggarwal et al. and George et al. have shown in their studies that high titers for B strains can be achieved after extensive optimization of moi, microcarrier concentration, agitation rate and CCI [10,47]. Thus, we are confident that additional optimization of CR.pIX cell-based processes (i.e. media and moi optimization) can further improve the propagation of the tested B/Vienna strain.

## Conclusions

Our study provides preliminary evidence for the potential for production of LAIV to high titers in a suspension process with an avian cell line. This process is fast with peak titers already 24 h post infection and easily scalable to industrial volumes as neither addition of microcarriers nor medium replacements are required. The use of an avian cell line designed specifically for vaccine production and a chemically-defined medium should facilitate regulatory approval. Optimization of medium, feeding or perfusion strategy and process parameters will be pursued to achieve a further increase in CCI and improvement of virus titers.

## Methods

### Cells

Suspension CR.pIX cells were routinely cultivated in 150 or 250 mL vented and baffled shaker flasks (working volume 50 or 150 mL, Corning) at 185 rpm and 5 cm amplitude or in T175 flasks (working volume 50 mL, Greiner) without agitation. Cultivation conditions were 37°C and 5% CO_2_ in chemically-defined medium (CD-U2, PAA) supplemented with glutamine, alanine (both 2 mM final concentration, Sigma) and recombinant insulin-like growth factor (LONG-R3 IGF, 10 ng/mL final concentration, Sigma). Cells were passaged twice a week by dilution with fresh medium.

### Virus strains

Wild type human influenza virus strain H1N1 A/PR/8/34 was received from the Robert Koch Institute (ampoule #3138) and adapted to growth in MDCK cells. Cold-adapted (*ca*) influenza virus strains were derived by cold-adaptation of master donor strains, reassortment with wild-type strains and subsequent adaptation to Vero cell cultures. These steps were done at Polymun Scientific GmbH according to Romanova et al. [11]. One A and one B strain of these Vero-adapted live attenuated viruses was used in the studies described here: human influenza H1N1 A/Singapore/1/57 *ca* × A/Singapore/2339/2000 (abbreviated as A/Singapore) and human influenza B/Vienna/1/1999/37 *ca* × B/Singapore/548/2000 (B/Vienna).

### Analytical methods

Cell concentration and viability were determined with a ViCELL XR device (BeckmanCoulter). Infectious titers (TCID_50_) were measured by titration of supernatants on MDCK monolayers. Samples from A/PR/8/34 experiments were analyzed at 37°C, whereas samples from LAIV strains were analyzed at 33°C. Antibodies against the HA protein were purchased from NIBSC institute, namely anti-A/PR/8/34 (NIBSC code #03/242), anti-A/Brisbane/59/2007 (NIBSC code #08/112) for detection of the A/Singapore strain and anti-B/Yamagata/16/88 (NIBSC code #92/582) for detection of the B/Vienna strain. All primary antibodies were used in a 1:200 dilution. As a secondary antibody we used donkey anti-sheep Alexa488 IgG (Invitrogen) in a 1:500 dilution. TCID_50_ titers (read-out: virions/mL) were calculated according to Spearman and Kärber [48]. Cell-specific virus yield was calculated by dividing the maximal infectious titers (from TCID_50_ assay) by the maximal viable cell concentration at or after time of infection. Samples from A/PR/8/34 experiments were additionally quantified by hemagglutination (HA) assay [49].

### Adaptation of virus strains to CR.pIX cells

CR.pIX-adapted working virus seeds were produced by passaging the Vero-adapted virus strains in T-flask cultures of CR.pIX cells (CCI 2 × 10^6^ cells/mL) using a moi of 10^-3^. Harvests from several passages and time points were titrated and harvests with highest titers were chosen retroactively as seed virus. Adaptation of the *ca* strains was done at a reduced temperature of 33°C. CR.pIX-adapted working virus seeds were stored in aliquots at −80°C for all further experiments.

### Small-scale infection experiments

After inoculating 8 × 10^5^ cells/mL, the cells were grown in shaker or T175 flasks up to 2 × 10^6^ cells/mL or highercell concentrations as indicated for the respective experiment. Calculated volumes of virus seed (for moi 10^-3^) and trypsin stock solution (500 units/mL) for the desired trypsin activity (as indicated for each experiment) were added directly to the cell suspension. In experiments where a complete medium exchange was performed, the whole culture was centrifuged at 150 × g for 10 min to collect cells prior to re-suspension in fresh medium. For a partial medium exchange, only half of the culture volume was centrifuged, re-suspended in fresh medium and subsequently pooled with the remaining non-centrifuged culture. Cultures were sampled once or twice per day. Cell concentration and viability were measured immediately; all samples for determination of virus titers (TCID_50_ and HA yields) were stored at −80°C until analysis of all samples belonging to the same experiment.

### Bioreactor infection experiments

Stirred tank bioreactors (1L working volume, DasGip AG) were inoculated with 8 × 10^5^ cells/mL. Monitoring and control of cultivations parameters were carried out with the cellferm-pro® system (DasGip AG). Bioreactors were equipped with pitched-blade stirrers operated at 120 rpm. The pO_2_ was set to 50% by pulsed aeration with air enriched with variable content of O_2_ and CO_2_. The pH value was allowed to drop from 7.4 to 7.0 during the first 72 h of cell growth and controlled at pH 7.0 afterwards. Analogous to infection optimization at small scale, cells were grown to the required cell concentration and subsequently infected by adding calculated volumes of A/Singapore virus (for moi 10^-3^) and a trypsin stock solution (5000 units/mL). Upon infection, cultivation temperature was reduced from 37°C to 33°C. All measurements were performed as described before.

## Competing interests

Patent applications covering avian cell lines (including AGE1.CR.pIX) have been filed by IJ and VS.

## Authors’ contributions

VL conceived the experiments and drafted the manuscript. SM conducted the adaptations of cold-adapted influenza virus strains and carried out preliminary experiments. IJ and YG edited the manuscript and participated in planning the experiments. DK and VS participated in designing the study. UR helped in drafting the manuscript and generally supervised the project. All authors read and approved the final manuscript.
